# Gas and Electricity

**Published:** 1910-08-27

**Authors:** H. B. Renwick

**Affiliations:** (General Manager and Secretary), The County of London Electric Supply Co., Ltd.


### GAS AND ELECTRICITY.

*To the Editor of* The Hospital.

Sir,—My attention has been called to a paragraph appearing in your issue of the 13th inst., relative to the contract recently entered into by the Finsbury Borough Council with the Gas Light and Coke Co. for the lighting of the Borough. You state that "this decision was taken after the latest examples of gas and electric lighting had been carefully inspected and their relative costs considered."

I am not in a position to say what were the latest examples of gas and electric lighting inspected by the Council, but I do know that my Company offered to submit examples of electric lighting free of expense to the Council, and this offer was declined. With regard to the question of relative running costs, my company submitted a tender for lighting which gave an illuminating power three times as great as the existing gas lighting, and moreover, showed a saving in running costs as compared with gas of over 14 per cent. In spite of this, however, the company's offer was declined. In view of the mistaken impression which the paragraph in question is likely to convey to your readers, I trust you will find space for this letter in your next issue.—Yours truly, H. B. Renwick.

(General Manager and Secretary),
The County of London Electric Supply Co., Ltd.
August 18, 1910.

